# Methods for simultaneously identifying coherent local clusters with smooth global patterns in gene expression profiles

**DOI:** 10.1186/1471-2105-9-155

**Published:** 2008-03-20

**Authors:** Yin-Jing Tien, Yun-Shien Lee, Han-Ming Wu, Chun-Houh Chen

**Affiliations:** 1 Institute of Statistics, National Central University, Tao-Yuan, 32001, Taiwan; 2 Genomic Medicine Research Core Laboratory, Chang Gung Memorial Hospital (CGMH), Tao-Yuan, 33305, Taiwan; 3 Department of Biotechnology, Ming Chuan University, Tao-Yuan, 33348, Taiwan; 4 Department of Mathematics, Tamkang University, Tamsui 25137, Taiwan; 5 Institute of Statistical Science, Academia Sinica, Taipei, 11529, Taiwan

## Abstract

**Background:**

The hierarchical clustering tree (HCT) with a dendrogram [1] and the singular value decomposition (SVD) with a dimension-reduced representative map [2] are popular methods for two-way sorting the gene-by-array matrix map employed in gene expression profiling. While HCT dendrograms tend to optimize local coherent clustering patterns, SVD leading eigenvectors usually identify better global grouping and transitional structures.

**Results:**

This study proposes a flipping mechanism for a conventional agglomerative HCT using a rank-two ellipse (R2E, an improved SVD algorithm for sorting purpose) seriation by Chen [3] as an external reference. While HCTs always produce permutations with good local behaviour, the rank-two ellipse seriation gives the best global grouping patterns and smooth transitional trends. The resulting algorithm automatically integrates the desirable properties of each method so that users have access to a clustering and visualization environment for gene expression profiles that preserves coherent local clusters and identifies global grouping trends.

**Conclusion:**

We demonstrate, through four examples, that the proposed method not only possesses better numerical and statistical properties, it also provides more meaningful biomedical insights than other sorting algorithms. We suggest that sorted proximity matrices for genes and arrays, in addition to the gene-by-array expression matrix, can greatly aid in the search for comprehensive understanding of gene expression structures. Software for the proposed methods can be obtained at .

## Background

Matrix visualization [4], for example the *Cluster and TreeView *package [5], is an important exploratory data analysis tool in the study of microarray gene expression profiles. The visual patterns of genes (rows) and arrays (columns) in the permuted gene-by-array expression profile matrix are useful for clustering purposes. The hierarchical clustering tree and the singular value decomposition are the two methods for identifying suitable gene/array permutations. This section briefly reviews the advantages and disadvantages of the two techniques using the fibroblast to serum gene expression data [1,6].

### Hierarchical clustering tree (HCT)

**Figure 1:**
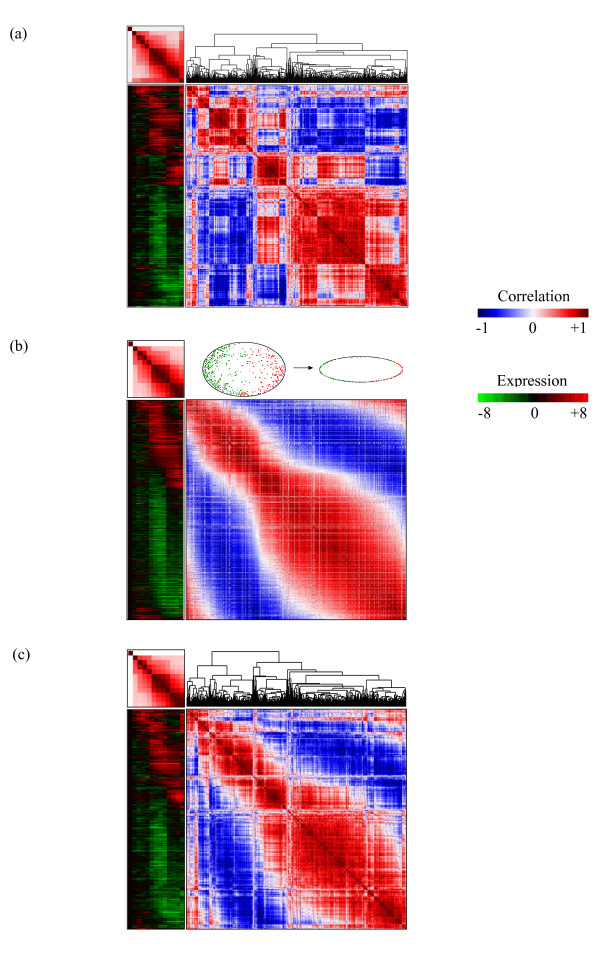
**Matrix visualization for expression profiles map with corresponding pair-wise correlation map for Fibroblast to serum data [1]. **Matrix visualization for expression profiles map with corresponding pair-wise correlation map for the time series of serum stimulation of primary human fibroblasts (Eisen *et al*. 1998) with three sorting algorithms. (a) Matrix visualization with hierarchical clustering tree (HCT). (b) Matrix visualization with rank-two ellipse seriation (R2E). (c) Matrix visualization with R2E guided HCT (HCT_R2E).

The dendrogram of an agglomerative hierarchical clustering tree (HCT) is constructed through a sequential bottom-up merging of "most similar" sub-nodes. This sequential mechanism guarantees good local grouping structures for permutations generated from rearranging terminal nodes of agglomerative HCTs. For a gene expression data matrix of 517 genes observed in 13 arrays (we use only the first 12 time series arrays, 0 minute to 24 hours) from the time series of serum stimulation of primary human fibroblasts, Eisen *et al*. [1] employed the Pearson product moment correlation to measure between-genes and between-arrays association. We adopt the average linkage option in calculating between-cluster relationships. We do not permute the array-array correlation matrix because of the time series nature of the 12 arrays, although the permuted result is identical to the original order for this particular correlation matrix. As illustrated in Figure 1a, an HCT is "grown" on the 517-by-517 correlation matrix for genes. The relative order of the 517 leaves of the dendrogram is then applied to sort the 517 rows/columns (symmetric) of the correlation matrix and the expression profile matrix.

The branching structure of a dendrogram plays an important role in identifying permutations of genes and arrays by its arrangement of intermediate nodes. For a given HCT with *n *terminal nodes (genes or arrays), there are *n*-1 intermediate nodes. Each of these intermediate nodes can be flipped independently resulting in 2^*n*-1 ^possible orderings of the terminal nodes from the same dendrogram built on the identical proximity matrix. Bar-Joseph *et al*. [7] had detailed discussion on the HCT intermediate nodes flipping phenomena. It was first formulated by Gruvaeus and Wainer [8]. To order the leaves of a binary HCT when two ordered branches are merged, the new branch is formed by placing the similar endpoints of the joining branches adjacent to each other. Many different heuristic ordering methods [1,9,10] have also been suggested for solving this problem. Bar-Joseph *et al*. [7] presented a fast optimal leaf ordering for the hierarchical clustering algorithm that maximizes the sum of the similarities of adjacent leaves in the Travelling Salesman sense [11], and we refer to this approach as the optimal tree method. Bar-Joseph *et al*. [12] proposed a heuristic algorithm for constructing *k*-ary trees by extending and improving the optimal leaf ordering algorithm in [7].

### Singular value decomposition (SVD) and Rank-two ellipse seriation (R2E)

For identifying smooth transitional expression patterns and more global-grouping structures, people turn to dimension reduction techniques, such as singular value decomposition, for help [2,13,14]. Alter *et al*. [2] laid down the mathematics of SVD for analyzing gene expression profiles and proposed the concept of eigenarrays and eigengenes as representative linear combinations of original arrays and genes. They further suggested that one sort the arrays and genes according to the relative positions on the subspaces spanned by the two leading eigenarrays and eigengenes.

Chen [3] introduced a sorting algorithm called rank-two ellipse (R2E) seriation which improves the SVD method by extracting the elliptical structure of the converging sequence of iteratively formed correlation matrices using the eigenvalue decomposition. Figure 1b displays the resulting matrix visualization of the human fibroblasts expression profile sorted by the R2E algorithm. We see that the R2E sorted correlation matrix identifies a very smooth transitional pattern. More advantages of the R2E method over the SVD method will be discussed in the Methods section.

### The proposed rank-two ellipse seriation-guided hierarchical clustering tree (HCT_R2E)

We propose to guide the flipping mechanism of a conventional agglomerative HCT using the rank-two ellipse (R2E) seriation of Chen [3] as an external reference. The resulting algorithm automatically integrates the desirable properties of HCT and R2E so that users have access to a clustering and visualization environment for gene expression profiles that preserves coherent local clusters and identifies global grouping trends.

The R2E-guided HCT with the corresponding permuted matrices can be seen in Figure 1c. The permuted correlation and gene expression matrices in Figure 1c resemble the corresponding matrices in Figure 1b extremely well, meaning that the coherent local structure (clusters) identified by the HCT architecture and the smooth global transitional pattern explored by the R2E algorithm do not necessarily conflict with each other. An important note here is that the dendrogram (hierarchical tree) architecture (merging steps) in Figure 1c (with R2E guide) is identical to that of Figure 1a (without R2E guide). The only thing different is the flipping mechanism of intermediate nodes.

### Global trend and the Robinson matrix

It is not common to permute the orders of arrays with time series nature for preserving the time-to-time local structure and the overall global time-trend. The local pattern and the global trend usually do not co-exist well in a given matrix unless a Robinson form [15] can be permuted from the matrix. A Robinson Matrix, *R *= [*r*_*ij*_], is a symmetric matrix such that *r*_*ij *_≤ *r*_*ik *_if *j *<*k *<*i *and *r*_*ij *_≥ *r*_*ik *_if *i *<*j *<*k*. The basic property of a Robinson matrix is monotonicity as one proceeds from the main-diagonal elements to all four margins of the given matrix. For a permuted proximity matrix, *D*_*n *× *n *_= [*d*_*ij*_], the following simple anti-Robinson loss function can be defined as the number of deviations from the Robinson form

(1) $$AR=\sum_{i=1}^{n}[\sum_{j<k<i}I(d_{ij}<d_{ik})+\sum_{i<j<k}I(d_{ij}>d_{ik})],$$

where *I *is an indicator function that outputs 1 if the condition is satisfied. More general anti-Robinson scores, generalized anti-Robinson (GAR), and relative generalized anti-Robinson (RGAR) scores, are defined in the Methods section.

**Figure 2:**
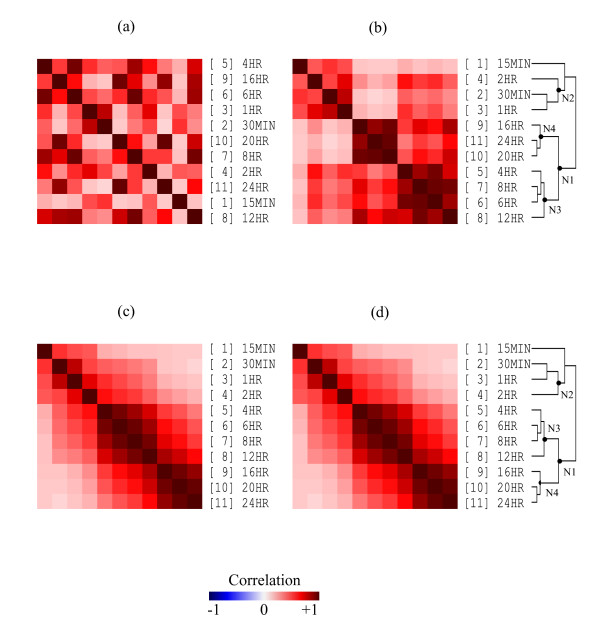
**Global trend and the Robinson matrix**. Matrix visualization for the between-array time series correlation matrix of data in [1]. (a) Between-array correlation matrix with time series randomized. (b) HCT sorted time series correlation matrix with local structure. (c) R2E sorted time series correlation matrix with (original) global trend. (d) HCT_R2E sorted time series correlation matrix preserves both (original) local and global patterns.

We elaborate on the global trend and the Robinson matrix concepts using the correlation matrix for the 11 arrays (15 min ~24 hr, 0 hr contains a constant array in the downloaded data) of the Eisen *et al*. [1] time series data. Figure 2a has the between-array correlation matrix with time points randomized. The up and down (non-monotonic) pattern in every column and row illustrates a clear non- (anti-) Robinson structure. Without a proper flipping guidance of the intermediate nodes, an HCT may end up as the matrix in Figure 2b. We can easily identify three local clusters (4–6–8–12 hr, 16–20–24 hr, and 0.25–0.5–1–2 hr) without observing the global smooth time series trend. The correlation matrix sorted by the R2E order (same as the original time order) keeps a near-Robinson form as can be seen in Figure 2c. When we guide the flipping mechanism of Figure 2b with the R2E global trend in Figure 2c, the permuted correlation matrix displays simultaneously the three-cluster pattern and the smooth time series trend, Figure 2d. For this particular example, the HCT_R2E permutation in Figure 2d happened to coincide with the R2E permutation in Figure 2c and the original time series order.

## Results

Three additional real data sets, together with the fibroblast to serum gene expression data, are analyzed to demonstrate the performance of the proposed method. The first one is the annotated subset cell cycle data from [16]; the second is the severe acute respiratory syndrome coronavirus (SARS-CoV) studied in [17]; the transition metal study in [18] is the final example. The same eight sorting algorithms (SVD with one eigenvector (SVD1), SVD with two eigenvectors (SVD2), self-organizing maps (SOM) [19], rank-two ellipse (R2E), HCT with random flips (HCT_RAM), optimal tree (HCT_OPT), SOM-guided tree (HCT_SOM), and R2E-guided tree (HCT_R2E)) are tested for all data sets. We only summarize the results of two HCT and two non-HCT algorithms: SVD2, R2E, HCT_OPT, and HCT_R2E. (Please see Additional file 1 for detailed comparison of all eight sorting algorithms.)

### Fibroblast to serum data

**Figure 3:**
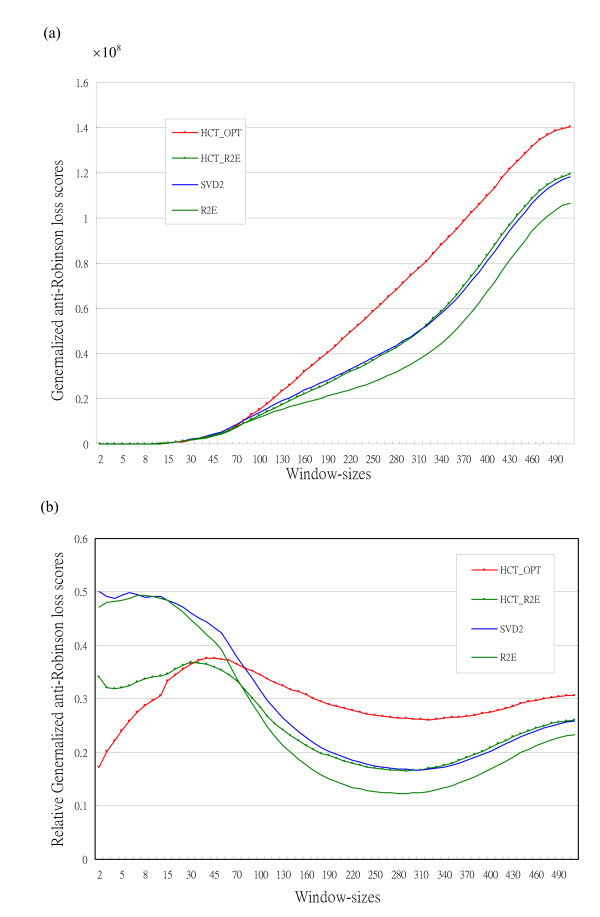
**Generalized anti-Robinson (GAR) loss scores for Fibroblast to serum data [1]**. Loss scores with varying window-sizes for correlation matrix with 517 genes data [1] for the sorting algorithms, SVD2, R2E, HCT_OPT and HCT_R2E. (a) Generalized anti-Robinson (GAR) loss scores. (b) Relative generalized anti-Robinson (RGAR) loss scores

For the gene expression data matrix of 517 genes observed in 12 arrays from the time series of fibroblasts to serum in [1], we plot the GAR loss scores and the RGAR loss scores in Figures 3ab without redrawing the permuted matrix visualizations.

### Results

The GAR curves (window-size ranges from 1 to 516) for the four sorting algorithms plotted in Figure 3a produce the following observations:

• the R2E (smooth green line) clearly outperforms (lowest GAR scores) the other three methods ;

• the HCT_OPT algorithm has poor global (large window-size) performance;

• the proposed HCT_R2E method outperforms HCT_OPT, and is nearly as good as the SVD2 algorithm in the global sense.

We plot in Figure 3b the relative generalized anti-Robinson (RGAR) loss scores for better comparison of local behaviours among the four methods, to observe the following:

• both HCT algorithms (curves with dots) outperform two non-HCT (smooth curves) in small window-size area (1 ≦ *w *≦ 50);

• the optimal hierarchical clustering tree, HCT_OPT, has the best performance among the four HCTs for the smallest window-size area (1 ≦ *w *≦ 35);

• the proposed HCT_R2E method actually scores best for a small period in the middle range (35 ≦ *w *≦ 75);

• the R2E algorithm dominates the competition from *w *= 100 on.

Without the visualization of two smooth transitional patterns for up- and down-regulated genes in Figure 1b, HCT in Figure 1a suggests many gene-clusters with very coherent expression profiles, but with no knowledge of the possible embedded smooth transitional patterns. The proposed HCT_R2E method automatically integrates the coherent local property of HCT and the smooth global trend of R2E to provide users the improved Figure 1c. The visualization of the expression profile and the correlation matrices in Figure 1c provide users exploration for local behaviour of genes function closely together in small time scale and for more complicate global relationship with larger time interval simultaneously in such a time series expression experiment.

### Yeast cell cycle data

These data are a subset of the original 6240 genes expressed at 17 time points used in Cho et al. [16]. We selected the 145 genes that have been biologically characterized and assigned to five different cell cycle phases (early G1, late G1, S, G2, and M). Expression at one abnormal time point was removed from the data set (as suggested by [20]) resulting in our gene expression profile of 145 genes at 16 time points.

### Results

**Figure 4:**
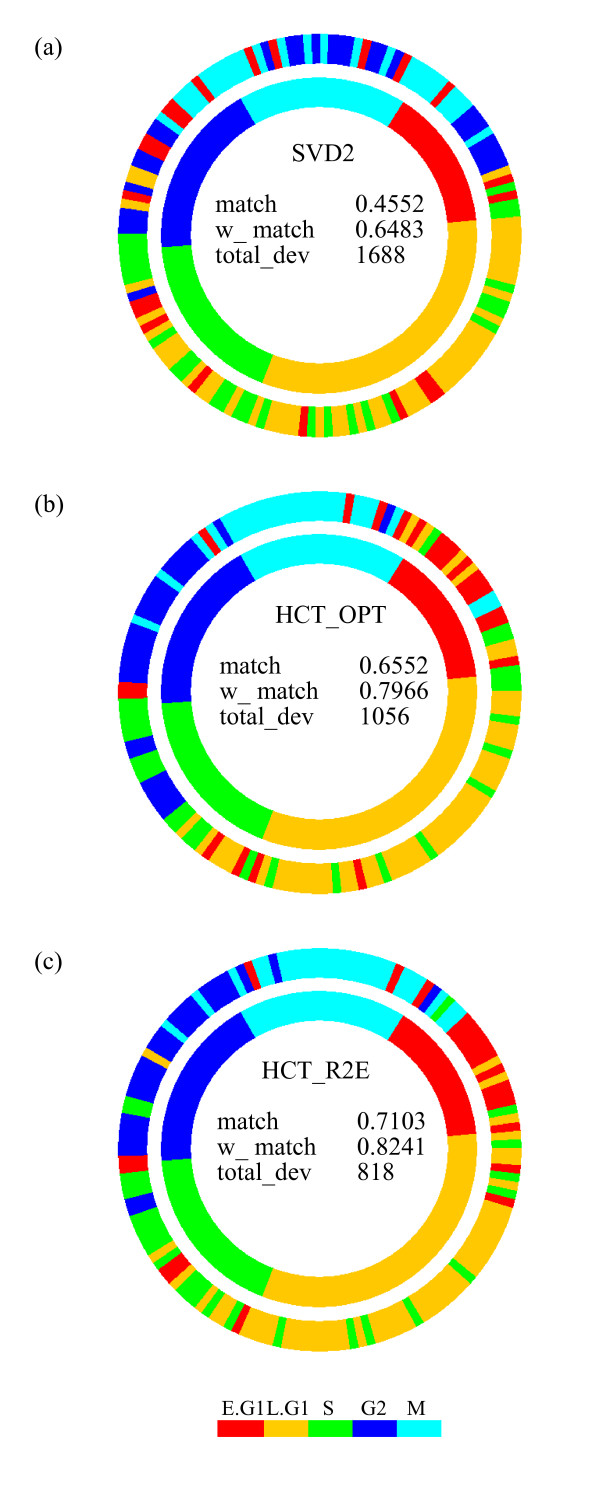
**Cell cycle phase diagrams for Yeast cell cycle data [16]**. Matching scores of the rearranged phase positions of the 145 genes sorted by the three seriation methods to the known (annotated) phase positions. (a) SVD2. (b) HCT_OPT order. (c) HCT_R2E order.

In addition to lower intermediate to global GAR and RGAR loss scores (see Additional file 1 for details), the permutation identified by the proposed HCT_R2E method also possesses more meaningful biological implications than the other algorithms. The cell cycle phase diagrams for the three seriation algorithms (SVD2, HCT_OPT, and HCT_R2E) are shown in Figure 4, where the identical inner circle represents the 145 genes sorted with the known cell cycle phase information. The outer circle for each algorithm is rotated to its best position among all 145 possible rotations according to the following criteria: the simple match score computes the proportion of correct (against known phase information) matches for all 145 gene positions, ranging from 0 (worst) to 1 (best); the weighted match score assigns weights of (2, 1, 0) to genes that deviate from the known phase by (0, 1, 2) phase groups, and is also scaled to 0 (worst) to 1 (best); the total deviation score sums the deviations (by number of genes) of all 145 genes to the boundaries of their known phases. Both the simple match and weighted match are gain scores (the higher the better) while the total deviation is a loss score (the lower the better).

From Table 1 we see that the proposed HCT_R2E algorithm outperforms the other seven algorithms in all three matching scores. Through visualization, the cell cycle diagrams sorted by the three algorithms can be roughly separated into three classes:

**Table 1:** Matching scores of the rearranged phase positions for the 145 genes sorted by the eight seriation methods relative to the known (annotated) phase positions.

Seriation method	Match	Weighted match	Total deviation
(a) SVD1	0.5103	0.6862	1584
(b) SVD2	0.4552	0.6483	1688
(c) SOM	0.6828	0.8068	907
(d) R2E	0.6759	0.8034	915
(e) HCT_RAM	0.5172	0.6759	1665
(f) HCT_OPT	0.6552	0.7966	1056
(g) HCT_SOM	0.5931	0.7379	1288
(h) HCT_R2E	**0.7103**	**0.8241**	**818**

• SVD2 performed rather poorly;

• HCT_OPT permutation showed better correlation to the known phases than SVD2;

• HCT_R2E arranged the 145 genes at positions very close to their annotated phase positions.

Although the HCT_R2E algorithm aligned the 145 genes close to their known phases, several genes deviated far away from their annotated cell cycle phases, as can be seen from the cell cycle diagram in Figure 4c. We further examined the phase annotations provided by another yeast cell cycle study of Spellman et al. [21]; the cross-annotated phase labels for both studies are listed in Additional file 2. The 15 genes with largest deviations from their annotated phase groups sorted by the proposed HCT_R2E algorithm are bold-faced. From the corresponding annotated phases of [21], in the last column, we see that the Spellman et al. [21] annotated phases for these 15 genes either fit better into the overall cell cycle pattern (e.g., YKL067W from S to G1, and YEL017W from early G1 to S/G2), or their phase conditions are not annotated (7 out of 15). This result further implies the proposed algorithm can be applied to either verify known biological conditions or to explore unknown phenomena.

### Severe acute respiratory syndrome coronavirus (SARS-CoV) data

In the severe acute respiratory syndrome (SARS) study of Lee *et al*. [17], the expression profiles of 52 signature genes are used to explore the between-sample severity pattern from normal controls to acute SARS patients. A Euclidean distance matrix among 55 samples (11 acute SARS (AS) patients, 33 recovering SARS (RS) patients, and 11 normal control (NC) subjects) using these 52 genes is computed to identify a potential order that could reflect the severity structure of the disease. There are three major differences between this SARS example and the yeast cell cycle data analysis. These are not time series gene expression data; the focus is on the between-sample structure instead of the gene set; and the proximity measure adopted is the between-sample Euclidean distance instead of the correlation coefficient.

### Results

**Figure 5:**
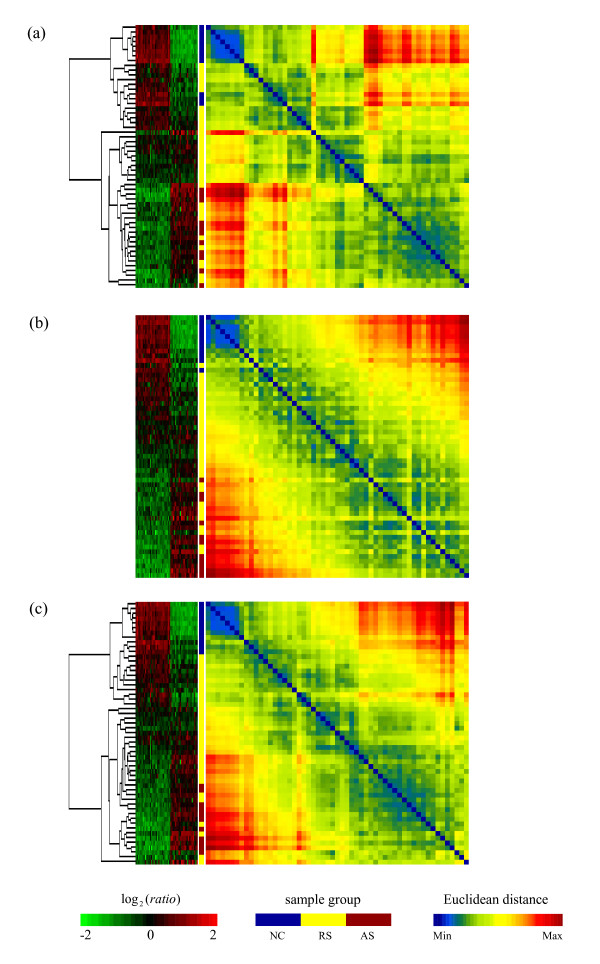
**Matrix visualization for Severe acute respiratory syndrome coronavirus (SARS-CoV) data [17]**. Matrix visualization for expression profiles map with corresponding pair-wise Euclidean distance map of 55 samples in the SARS study [17] with three sorting algorithms. (a) HCT_OPT order. (b) R2E seriation. (c) HCT_R2E order.

The same eight algorithms are used to sort the Euclidean distance matrices for the 55 samples but only results of the three methods, HCT_OPT, R2E, and HCT_R2E are displayed. The corresponding expression profile matrices with related HCT dendrograms and the sorted colour bands for sample identities are displayed in Figure 5. We observe the following:

• there is a clear uni-dimensional Robinson pattern for this SARS Euclidean matrix;

• the HCT_OPT (Figure 5a) algorithm presented rather coherent local structure;

• R2E (Figure 5b) sorted samples identify colour bands that exhibit a clear blue (NC) to yellow (RS) to red (AS) severity structure of the disease;

• the Euclidean matrix sorted by the proposed HCT_R2E (Figure 5c) method displays very coherent local relationships, as well as extremely good global structure. Its identity colour band has a coherent within sample-subtype pattern

We have summarized the numerical comparisons (GAR, RGAR) for the eight sorting algorithms in Additional file 1.

In [17], the R2E permuted sample rank of SARS severity was identified to be significantly correlated with the clinical pulmonary infection score (CPIS) and other clinical factors. The severity rank of samples was also found to be highly correlated with the suppression of the human adaptive immune system and the up-regulation of the host receptors for corona viruses of SARS. Here we compare the Pearson correlations (Spearman and Kendall correlations give similar results) of the patient-orders identified by the eight algorithms with two clinical variables: number of days after the onset of disease and the clinical pulmonary infection score (CPIS). The results are summarized in Table 2 and we note the following:

**Table 2:** Correlations between the SARS severity ranks derived from eight seriation methods using number of days after the onset of disease and clinical pulmonary infection score (CPIS).

Seriation method	Pearson correlation (days)	Pearson correlation (CPIS)
(a) SVD1	0.6303	0.5006
(b) SVD2	0.3276	0.1873
(c) SOM	0.4028	0.2925
(d) R2E	0.6497	0.4890
(e) HCT_RAM	0.1551	0.0230
(f) HCT_OPT	0.4249	0.5006
(g) HCT_SOM	0.6468	0.3151
(h) HCT_R2E	**0.6693**	**0.5116**

• the proposed HCT_R2E algorithm has the highest correlation with number of days after the onset of disease while the R2E method comes next;

• the proposed HCT_R2E algorithm has the highest correlation with CPIS among all eight sorting methods, while the SVD1 and HCT_OPT algorithms share second place

From these comparisons we observe a significant advantage of the proposed R2E-guided hierarchical clustering tree in searching for meaningful biomedical information and correlation such that researchers can further propose more precise hypotheses and conducting more accurate experiments.

### Transition metal stress data

Kaur *et al*. [18] tried to reconstruct physiological behaviours of *Halobacterium NRC-1*, an archaeal halophile, in sublethal stress levels of six transition metals (Mn [II], Fe [II], Co [II], Ni [II], Cu [II], and Zn [II]). *Halobacterium NRC-1 *was exposed for five hours to at least three concentrations of each of the six transition metals. In Figure 5 of [18], using 468 genes that changed significantly in at least two conditions out of a total of 19 (3 concentrations for each of the 6 transition metal with an additional concentration from Fe [II]), an HCT and a correspondence analysis (CA, [22]) are carried out (we only obtained 444 genes using identical selection criteria). Their HCT permutation for the 19 metal conditions does not correlate well with the pattern displayed in their CA plot for the conditions. Our task here is to guide the flips of HCT intermediate nodes by the R2E algorithm with the hope that the resulting permutation does not contradict that of the CA analysis.

### Results

**Figure 6:**
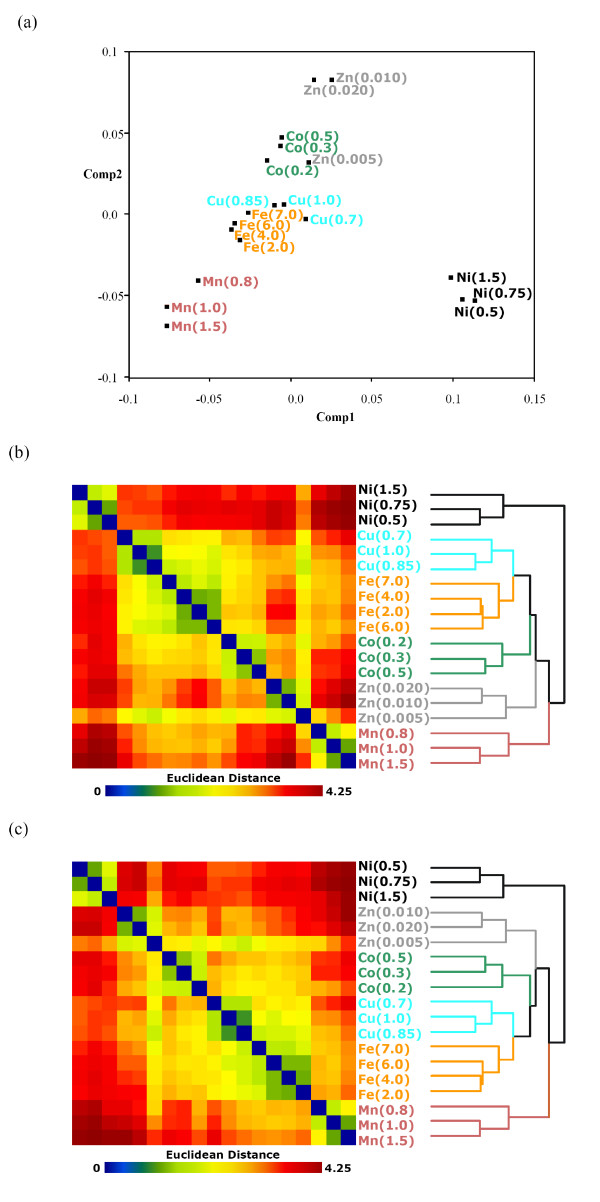
**Transition metal stress data [18]**. Visualization of differential gene expression profiles of *Halobacterium NRC-1 *exposed for five hours to at least three concentrations of each of the six transition metals (Mn [II], Fe [II], Co [II], Ni [II], Cu [II], and Zn [II]). (a) Correspondence analysis of the 19 metal-concentration combinations. (b) HCT_OPT with sorted Euclidean matrix map for the 19 conditions. (c) HCT_R2E with sorted Euclidean matrix map for the 19 conditions.

The CA plot is reconstructed in Figure 6a. Information for the 444 genes is not displayed for better illustration of the 19 metal conditions. A clear linear trend Mn [II]-Fe [II]-Cu [II]-Co [II]-Zn [II] is observed, with Ni [II] conditions that deviate significantly from this trend. An average linkage HCT is built on the paired Euclidean distance matrix of the 19 metal conditions ([18] did not specify proximity measure and linkage type in their study). The optimal HCT and the proposed elliptical seriation-guided HCT with their permuted Euclidean matrices are displayed in Figure 6bc. Although HCT_OPT does identify good local clusters for the metal groups, the overall permutation does not correlate well with the linear trend from the CA analysis. The HCT_R2E permutation not only correlates with the linear trend of transition metal groups very well, it also sorts the within-metal group concentration levels precisely following those orders in the CA analysis in Figure 6a.

This study illustrates well that the proposed HCT_R2E method is capable of providing permutations with both good global and local properties, although the optimal HCT still outputs better local orders numerically. The accompanying distance matrix map clearly indicates the Zn(0.005) and Cu(0.7) conditions, in addition to the Ni [II] conditions, deviate from the main linear trend of these transition metals and the Robinson pattern.

## Discussion and Conclusion

When analyzing gene expression profile data sets, researchers usually apply a hierarchical clustering tree (HCT) to search for coherent local clusters and the singular value decomposition (SVD) to identify smooth global trends. Users of HCT dendrograms would identify only local clusters without knowing the existence of global structure that might accompany cell cycle-regulated experiments, dosage level studies, or subtypes of tumours. Applications of SVD on the other hand may overlook the importance of local behaviour.

While the optimal HCT [7] always produces permutations with best local behaviour, the rank-two ellipse seriation [3] gives the best global grouping patterns and smooth transitional trends. The proposed hierarchical clustering tree guided by rank-two ellipse seriation (HCT_R2E) nicely integrates these two extremes and provides users both coherent local clusters and smooth global patterns for gene expression profile studies.

In four data analyses, the proposed HCT_R2E algorithm not only exhibits outstanding numerical (statistical) performance, it also provides us better insights into the biomedical information embedded in these high dimensional data structures. Visualization of sorted proximity matrices in addition to the visualization of the expression profile matrices also greatly enhances the overall comprehension of the association structures of arrays and genes.

### Applicability and limitation

As was illustrated in the two time series data sets, the proposed rank-two ellipse-guided hierarchical clustering (HCT_R2E) is very powerful in identifying smooth time series patterns. The SARS data and the transitional metal data, on the other hand, showed the proposed method can also be used to search for potential global grouping structure for genes, and for arrays embedded in the given gene expression profiles.

When the underlying clustering pattern is a clear disjoint one, the rank-two ellipse seriation method is only capable of identifying the global between-cluster pattern, not the within-cluster relationship. The optimal tree method gives better permutations than the proposed method for such circumstances.

The R2E algorithm (and the HCT_R2E method) is computationally more time consuming than other methods. It takes a personal computer (Celeron (R) 3.2 GHz CPU with 512 MB RAM) running C++ on Windows XP about (0.09 sec, 9.09 sec, and 2.71 hr) to obtain the R2E permutations for proximity matrices with (50, 500, 5000) rows/columns. The computation complexity for R2E is of order n^3^. The computing speed is much slower in the current Java version GAP package although we are implementing a much faster algorithm now. We have also developed a prototype PC cluster system for performing the proposed methods for very large proximity matrices that will be released after it has been fully tested.

## Methods

Various concepts have been proposed for rearranging objects in statistical graphs in order to display information structure more effectively. Chen [3] proposed the concept of "relativity of a statistical graph" for placing similar (different) objects at closer (distant) positions in a statistical graph. The local property optimized by the aforementioned HCT techniques realizes only half of the relativity concept when it places similar objects in closer proximity without the necessity of distancing distinct objects.

### Rank-two ellipse seriation

Chen [3] introduced a sorting algorithm called rank-two ellipse (R2E) seriation that extracts the elliptical structure at iteration with rank two of the converging sequence of iteratively formed correlation matrices. R2E improves SVD in identifying even smoother global permutations. Please see Additional file 1 for an illustration with the 517 gene example. The permuted expression profile matrix and the sorted gene-by-gene correlation matrix using R2E are displayed in Figures 1b. The sorted expression matrix displays a clear smooth transitional two-component pattern.

There are two advantages of the R2E method over the SVD method in the sorting of arrays and genes in expression profile matrices. The first is that users do not need to choose the number of leading components; the R2E method always summarizes the embedding variation structure into the final two eigenvectors of the rank-two correlation matrix. With a uni-dimensional underlying structure, the two eigenvectors form a half-ellipse pattern for sorting purposes. The second advantage is that it can be applied to any given proximity matrix, be it correlation, covariance, Euclidean distance, or other proximity matrix for genes and arrays.

### Proximity matrix visualization

Although both the dendrogram of an HCT and representative genes (arrays) of an SVD are generated from given proximity matrices, researchers usually do not pay much attention to the sorted proximity matrices.

Comparing the permuted gene-by-gene correlation matrices in Figures 1a and 1c we see that the HCT forms many blocks along the main diagonal of the correlation matrix while rank-two method identifies two smooth transitional patterns for up- and down-regulated genes. Without the visualization of correlation matrix in Figure 1a, HCT suggests many gene-clusters with very coherent expression profiles, but with no knowledge of the possible embedded smooth transitional patterns. In light of both correlation matrices in Figures 1a and 1c one can see that the gene-clusters actually are formed only because of the constraints imposed by the HCT dendrogram branching structure; the within-cluster coherent expression profiles are correctly identified, but the between-clusters contrasting patterns may not be applicable.

In addition to the visualization of permuted expression profile matrices, we want to emphasize the importance of visualization of sorted proximity matrices for comparing the differences in permutations that result from various sorting algorithms.

### Integration of local clustering patterns and global grouping structures

Local coherent gene clusters with very similar expression profiles may represent groups of genes that are co-regulated by certain transcription factors or activated by identical binding sites. Global clustering patterns and smooth transitional trends on the other hand, could signal some biological processes at a higher-level control, such as metabolite pathways or the cell-cycle operation. It is necessary to develop clustering and visualization methods that can simultaneously explore local behaviours as well as global grouping effects of gene expression profiles.

**Figure 7:**
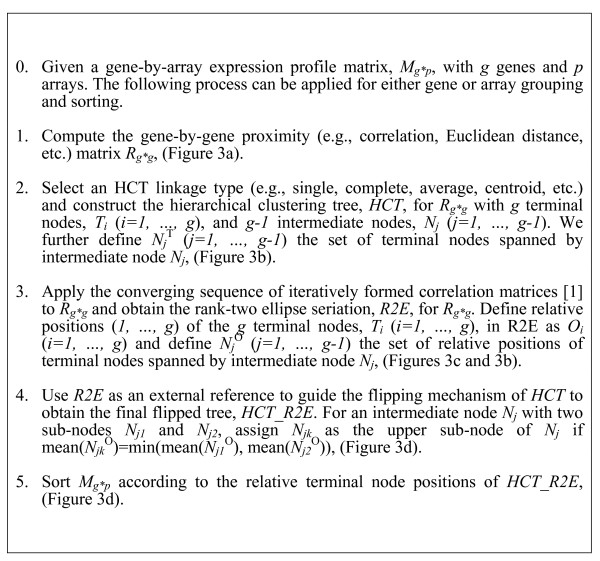
**Proposed R2E guided HCT procedure for gene clustering**. The proposed algorithm for constructing the R2E-guided HCT for gene permutation using the between array correlation matrix in Figure 2.

This study proposes to guide the flipping mechanism of a conventional agglomerative HCT with the rank-two ellipse (R2E) seriation as an external reference. The standard working procedure of the proposed algorithm for gene clustering is illustrated as steps 0~5 in Figure 7, using Figure 2 as an example. The same process can be applied for array grouping and sorting.

We use the intermediate node N_1 _with sub-nodes (N_3 _and N_4_) in Figure 2b to illustrate the flipping mechanism described in steps 2~4 of Figure 7. The external reference R2E identified in Figure 2c assigned the relative positions (1~11) to the 11 terminal nodes of Figure 2b in square brackets. We have *N*_3_^T ^= {4HR, 6HR, 8HR, 12HR} and *N*_4_^T ^= {16HR, 20HR, 24HR} with *N*_3_^O ^= {5, 6, 7, 8} and *N*_4_^O ^= {9, 10, 11}. N_3 _and N_4 _are assigned the upper and lower sub-nodes of N_1 _respectively since mean(*N*_3_^O^) = 6.5 < 10 = mean(*N*_4_^O^).

### Generalized anti-Robinson criteria

**Figure 8:**
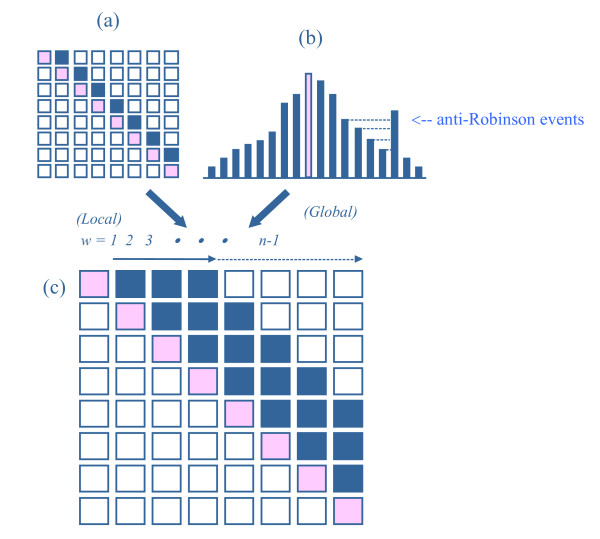
**Generalized anti-Robinson criteria**. Three anti-Robinson concepts. (a) Local traveling salesman criterion. (b) Global Robinson concept. (c) Combined generalized anti-Robinson criterion.

In order to compare the performances of different sorting algorithms, some standard criteria have to be established. As is illustrated in Figure 8a, the minimum travelling distance in a travelling salesman problem can be used to evaluate local behaviour, while the anti-Robinson event-count (*AR *in equation 1 and Figure 8b) works well for global performance. Given a distance-type proximity matrix, the travelling salesman algorithm optimizes the permutation by minimizing the total consecutive distances along the entire permutation. That is, one minimizes the summation along the off-diagonal containing the *i*th to (*i+1*)st components of the matrix (Figure 8a).

For a permuted proximity matrix, *D*_*n *× *n *_= [*d*_*ij*_], the generalized anti-Robinson loss function is defined as the number of deviation from the Robinson form,

(2) $$GAR=\sum_{i=1}^{n}[\sum_{(i-w)\leq j<k<i}I(d_{ij}<d_{ik})+\sum_{i<j<k\leq (i+w)}I(d_{ij}>d_{ik})],$$

where *w *is the window-size defining the range of summation, and *I *is an indicator function that outputs 1 if the condition is satisfied. Window-size is the number of columns (rows) from the diagonal of D that we consider in calculating the anti-Robinson events. Small window-sizes refer to criteria for considering only local behaviours, and larger window-sizes refer to criteria for more global relationship between subjects.

The minimum travelling distance can be treated as one special Robinson form with a smallest window-size (*w *= 1) in counting the anti-Robinson events, while the original *AR *(equation 1) criterion has the largest window-size (*w *= *n *- 1). A window-size between 1 and *n-*1 opens up a banding area from the main-diagonal for counting the number of anti-Robinson events. This is called the generalized anti-Robinson criterion (GAR) here. When we plot the GAR scores against *w *(window-size) we usually see a monotonic smooth increasing curve since the number of anti-Robinson events grows larger with window-size. In order to have better comparison among different sorting algorithms for small window-sizes we also define the relative generalized anti-Robinson loss function,

(3) $$RGAR=\frac{\sum_{i=1}^{n}[\sum_{(i-w)\leq j<k<i}I(d_{ij}<d_{ik})+\sum_{i<j<k\leq (i+w)}I(d_{ij}>d_{ik})]}{\sum_{i=1}^{n}[\sum_{(i-w)\leq j<k<i}1+\sum_{i<j<k\leq (i+w)}1]},$$

which ranges between 0 (no anti-Robinson events) to 1 (all anti-Robinson events). The *RGAR *curves have better resolution for small window-size region than the *GAR *curves for comparing performance of algorithms.

## Availability and requirements

The rank-two ellipse (R2E) seriation and the R2E-guided hierarchical clustering tree methods are implemented in the GAP (generalized association plots) system.

Project name: HCT-R2E

Project home page: 

Operating systems: any OS that supports the Java environment

Programming language: Java

License: free

## Authors' contributions

YJT and CHC conceived the study, developed the methodology, and wrote the manuscript. YSL performed the data analysis. HMW implemented the HCT-R2E algorithm and the GAP environment. All authors read and approved the final manuscript.

## Supplementary Material

Additional File 1 Supplementary_material. The zipped archive [supplementary_material.zip] contains 1 documentary file (supplement.doc) and 8 Figure files (Figure S1–8) as the supplement for this main article. It has more detailed information about matrix visualization and numerical comparisons of 8 sorting algorithms for Fibroblast to serum data, Yeast cell cycle data, Rank-two ellipse seriation, the figures for sequence of R2E convergency patterns, and SARS-CoV data with an introduction to the GAP software. Click here for file

Additional File 2 Cell cycle phase annotations. The zipped archive [cell_cycle.zip] contains 1 Excel file (cell_cycle_phase_annotations.xls). Cell cycle phase annotations of the 145 genes in Cho *et al*. [16] cross-annotated by Spellman et al. [21]. Genes are arranged by the proposed HCT_R2E algorithm; phase conditions for [16] are colour coded according to the phase legend provided in Figure 4. Click here for file
