# Supplementary material for: Methods for simultaneously identifying coherent local clusters with smooth global patterns in gene expression profiles
Source: BMC Bioinformatics. 2008 Mar 20;9:155. doi: 10.1186/1471-2105-9-155 (PMC2322988; doi:10.1186/1471-2105-9-155)
Supplement: Additional File 1 — Supplementary_material. The zipped archive [supplementary_material.zip] contains 1 documentary file (supplement.doc) and 8 Figure files (Figure S1–8) as the supplement for this main article. It has more detailed information about matrix visualization and numerical comparisons of 8 sorting algorithms for Fibroblast to serum data, Yeast cell cycle data, Rank-two ellipse seriation, the figures for sequence of R2E convergency patterns, and SARS-CoV data with an introduction to the GAP software. [file 1471-2105-9-155-S1.zip › FigureS4.doc]

SVD1

match	0.5103

w_match	0.6862

total_dev	1584

(a)

(e)

HCT_RAM

match	0.5172

w_ match	0.6759

total_dev	1665

(b)

(f)

SVD2

match	0.4552

w_ match	0.6483

total_dev	1688

HCT_OPT

match	0.6552

w_ match	0.7966

total_dev	1056

(c)

(g)

SOM

match	0.6828

w_ match	0.8068

total_dev	907

HCT_SOM

match	0.5931

w_ match	0.7379

total_dev	1288

E.G1 L.G1	S	G2	M

(d)

(h)

R2E

match	0.6759

w_ match	0.8034

total_dev	915

HCT_R2E

match	0.7103

w_ match	0.8241

total_dev	818
