# Supplementary material for: Methods for simultaneously identifying coherent local clusters with smooth global patterns in gene expression profiles
Source: BMC Bioinformatics. 2008 Mar 20;9:155. doi: 10.1186/1471-2105-9-155 (PMC2322988; doi:10.1186/1471-2105-9-155)
Supplement: Additional File 1 — Supplementary_material. The zipped archive [supplementary_material.zip] contains 1 documentary file (supplement.doc) and 8 Figure files (Figure S1–8) as the supplement for this main article. It has more detailed information about matrix visualization and numerical comparisons of 8 sorting algorithms for Fibroblast to serum data, Yeast cell cycle data, Rank-two ellipse seriation, the figures for sequence of R2E convergency patterns, and SARS-CoV data with an introduction to the GAP software. [file 1471-2105-9-155-S1.zip › FigureS5.doc]

(f)

(c)

(h)

(d)

(g)

(b)

(e)

(a)

sample group	Euclidean distance

-2	0	2	NC	RS	AS	Min	Max
