# Supplementary material for: Methods for simultaneously identifying coherent local clusters with smooth global patterns in gene expression profiles
Source: BMC Bioinformatics. 2008 Mar 20;9:155. doi: 10.1186/1471-2105-9-155 (PMC2322988; doi:10.1186/1471-2105-9-155)
Supplement: Additional File 1 — Supplementary_material. The zipped archive [supplementary_material.zip] contains 1 documentary file (supplement.doc) and 8 Figure files (Figure S1–8) as the supplement for this main article. It has more detailed information about matrix visualization and numerical comparisons of 8 sorting algorithms for Fibroblast to serum data, Yeast cell cycle data, Rank-two ellipse seriation, the figures for sequence of R2E convergency patterns, and SARS-CoV data with an introduction to the GAP software. [file 1471-2105-9-155-S1.zip › supplement_20080311doc.doc]

**Supplementary material to Tien Lee Wu and Chen 2008**

## Fibroblast to serum data [1]

For the gene expression data matrix of 517 genes observed in 12 arrays from the time series of fibroblasts to serum, we plot the GAR loss scores and the RGAR loss scores in Figures S1ab without redrawing the permuted matrix visualizations.

The GAR curves (window-size ranges from 1 to 516) for the eight sorting algorithms plotted in Figure S1a produce the following observations:

- the R2E (smooth green line) clearly outperforms (lowest GAR scores) all other seven methods;
- SVD1 has the overall worst performance;
- the random flipping HCT, HCT_RAM is the worst HCT among four hierarchical clustering tree algorithms (curves with dots);
- the HCT_OPT algorithm has poor global (large window-size) performance;
- the proposed HCT_R2E method outperforms the other three HCTs, and is nearly as good as the SVD2 and SOM algorithms in the global sense.

We plot in Figure S1b the relative generalized anti-Robinson (RGAR) loss scores for better comparison of local behaviours among the eight methods, to observe the following:

- all four HCT algorithms (curves with dots) outperform all four non-HCT (smooth curves) in small window-size area (1≦*w*≦50);
- the optimal hierarchical clustering tree, HCT_OPT, has the best performance among the four HCTs for the smallest window-size area (1≦*w*≦35);
- the SOM-guided HCT, HCT_SOM, performs worst among the four HCT methods for this region, even worse than the randomized tree;
- the proposed HCT_R2E method actually scores best for a small period in the middle range (35≦*w*≦75);
- the R2E algorithm dominates the competition from *w* =100 on.

## Matrix Visualization and numerical comparison of 8 sorting algorithms for Yeast cell cycle data [2]

The permuted expression profile matrices and correlation matrices for 145 genes, with corresponding colour bands for phases of cell cycle using eight different algorithms, are displayed in Figure S2. We have summarized the following observations:

- there is no doubt that the R2E algorithm identifies the best global transitional expression pattern (Figure S2d) among all eight algorithms;
- the R2E permuted gene-by-gene correlation matrix (Figure S2d) also has a near Robinson pattern with very smooth transitional correlation structure, except for the upper-right (and lower-left) corner which exhibits a bridging effect between the early G1 and late G1 phases;
- the R2E permuted cell cycle phase band reveals a coherent pattern of (late G1, S, G2, M, early G1);
- although the SOM permuted expression and correlation matrices (Figure S2c) also have better global structures, they are not as smooth as those of R2E;
- both SVD1 and SVD2 algorithms have poor local and global performance;
- all four HCT-related permutations in Figures S2 e, f, g, and h exhibit significant anti-Robinson branching effects;
- from the darker red banding along the diagonal, one can see that the HCT_OPT algorithm (Figure S2f) has tightest local behaviour;
- the proposed HCT_R2E method (Figure S2h), while preserves the local behaviour of HCT_OPT, has better global transitional pattern than the HCT_OPT method. The proposed HCT_R2E method also maintains the coherent (late G1, S, G2, M, early G1) cell cycle phases of R2E algorithm.

The detailed numerical comparisons of GAR loss scores (window-size ranges from 1 to 144) for all six algorithms are shown in Figure S3a with the following remarks:

- R2E has the best overall performance, followed by SOM, among all six algorithms;
- the proposed HCT_R2E method has best mid-range to global performance among all four HCT algorithms;
- the SVD1 and SVD2 algorithms performed very poorly except at extremely global window-sizes. There they outperformed the R2E algorithm because of the bridging effect between the early G1 and late G1 phases of the R2E method.

We further plot the relative generalized anti-Robinson (RGAR) loss scores for the eight methods in Figure S3b, with the following findings:

- all four HCT algorithms (curves with dots) outperform all four non-HCT (smooth curves) in small window-size area (1≦*w*≦30);
- the HCT_OPT algorithm has the best local results for (1≦*w*≦27)
- the SOM method has the best mid-range results for (28≦*w*≦60);
- The proposed HCT_R2E method outperforms the other three HCT algorithms for mid-range to global region (38≦*w*≦144).

The results of the analyses of the cell cycle data are as expected. The HCT_OPT permutation had the best local performance, in the travelling salesman sense, while the R2E seriation gave the best global pattern using the Robinson metric. The proposed rank-two ellipse seriation-guided hierarchical clustering tree preserved the nice properties of these two extremes, and maintained coherent local clusters as well as smooth global transitional trends.

The cell cycle phase diagrams for the eight seriation algorithms are shown in Figure S4, where the identical inner circle represents the 145 genes sorted with the known cell cycle phase information. The outer circle for each algorithm is rotated to its best position among all 145 possible rotations according to the following criteria: the simple match score computes the proportion of correct (against known phase information) matches for all 145 gene positions, ranging from 0 (worst) to 1 (best); the weighted match score assigns weights of (2, 1, 0) to genes that deviate from the known phase by (0, 1, 2) phase groups, and is also scaled to 0 (worst) to 1 (best); the total deviation score sums the deviations (by number of genes) of all 145 genes to the boundaries of their known phases. Both the simple match and weighted match are gain scores (the higher the better) while the total deviation is a loss score (the lower the better).

## Matrix Visualization and numerical comparison of 8 sorting algorithms for Severe acute respiratory syndrome coronavirus (SARS-CoV) data [3]

The same eight algorithms are used to sort the Euclidean distance matrices for the 55 samples. The corresponding expression profile matrices with related HCT dendrograms and the sorted colour bands for sample identities are displayed in Figure S5. We observe the following:

- there is a clear uni-dimensional Robinson pattern for this SARS Euclidean matrix;
- the SVD1 (Figure S5a) and R2E (Figure S5d) sorted samples both identify colour bands that exhibit a clear blue (NC) to yellow (RS) to red (AS) severity structure of the disease;
- the SVD2 (Figure S5b) and SOM (Figure S5c) algorithms did poorly on both local and global scales;
- the Euclidean matrix sorted by the proposed HCT_R2E (Figure S5h) method displays very coherent local relationships, as well as extremely good global structure. Its identity colour band has a coherent within sample-subtype pattern.

The detailed numerical comparisons of GAR and RGAR loss scores (window-size ranges from 1 to 54) for all eight algorithms are given in Figures S6 ab for the SARS data with the following observations:

- SVD1 has an almost identical performance as that of the R2E algorithm;
- all four HCT algorithms as usual outperform all four non-HCT in small window-size area (1≦*w*≦12);
- SVD2 did poorly overall;
- the proposed HCT_R2E algorithm has a very good overall performance, nearly as well as those of SVD1 and R2E even for global (large window-size) region;
- there is a large margin between the group (SVD1, R2E, and HCT_R2E) and the others;
- the best performance belong to three different algorithms over three regions of window-sizes: HCT_OPT for 1<w <10; HCT_R2E for 10≦*w*≦22; R2E and SVD1 for 22≦w.

## Rank-two ellipse seriation

Chen [4] introduced a sorting algorithm called rank-two ellipse (R2E) seriation that extracts the elliptical structure of the converging sequence of iteratively formed correlation matrices using eigenvalue decomposition. As illustrated in Figure S7, the converging sequence of the correlation matrices gradually merges relative information structure of the minor (3^rd^ and beyond) eigenvectors into the leading two vectors. At the beginning of the iterating process (iteration 1), this method is nothing but the eigenvalue decomposition of the original correlation matrix and a large portion of information of the correlation matrix is still contained in the 3^rd^ and beyond eigenvectors. Throughout the converging process, the information accompanying these eigenvectors is merged (reduced) into the leading two eigenvectors. At the iteration with rank two, there are only two eigenvectors left with non-zero eigenvalues, and information is reduced to the ellipse spanned by the first two eigenvectors. Every gene (or array) has its relative position on this two-dimensional ellipse, and a unique permutation is obtained. At the end of the converging process, eventually all information is reduced into the first eigenvector and the 517 genes are split into two disjoint groups, the relatively up-regulated (red) and down-regulated (green) gene clusters.

## GAP software

The proposed HCT_R2E method has been implemented in a Java-designed software packages, GAP (generalized association plots) - an interactive graphical environment for information visualization and exploratory data analysis (EDA, [5]). GAP integrates visualization of the data matrix with two proximity matrices for extracting information embedded in these matrices. Various clustering methods with matrix sorting algorithms are implemented. GAP provides users a simple yet powerful tool for visually interacting with the matrices and exploring the structure embedded in high-dimensional data matrices before formal mathematical operations and statistical modelling can be carried through for a more comprehensive data analysis. GAP is available to registrants and is free to non-commercial applications. The software, user manual and tutorials can be obtained at [http://gap.stat.sinica.edu.tw/Software/GAP. Figure S8](http://gap.stat.sinica.edu.tw/Software/GAP. Figure S4) illustrates part of the working environment for GAP using SARS-CoV data with the proposed HCT_R2E procedure as an example.

# References

1. Eisen MB, Spellman PT, Brown PO, Botstein D: **Cluster analysis and display of genome-wide expression patterns.** *PNAS* 1998, **95**: 14863-14868
2. Cho RJ, Campbell MJ, Winzeler EA, Steinmetz L, Conway A, Wodicka L, Wolfsberg TG, Gabrielian AE, Landsman D, Lockhart DJ, Davis RW: **A genome-wide transcriptional analysis of the mitotic cell cycle.** *Mol. Cell* 1998, **2**: 65-73
3. Lee YS, Chen CH, Chao A, Chen ES, Wei ML, Chen LK, Yang K, Lin MC, Wang YH, Liu JW, Eng HL, Chiang PC, Wu TS, Tsao KC, Huang CG, Tien YJ, Wang TH, Wang HS, Lee YS: **Molecular signature of clinical severity in recovering patients with severe acute respiratory syndrome coronavirus (SARS-CoV).** *BMC Genomics* 2005, **6**: 132
4. Chen CH: **Generalized association plots for information visualization: The applications of the convergence of iteratively formed correlation matrices.** *Statistica Sinica* 2002, **12**: 1-23
5. Tukey JW: **Exploratory Data Analysis.** Addison-Wesley.

**Supplement figures**

**Figure S1 - Generalized anti-Robinson (GAR) loss scores for Fibroblast to serum data [1]**

Loss scores with varying window-sizes for correlation matrix with 517 genes data [1] for the sorting algorithms, SVD1, SVD2, SOM, R2E, HCT_RAM, HCT_OPT, HCT_SOM, and HCT_R2E.

(a) Generalized anti-Robinson (GAR) loss scores
(b) Relative generalized anti-Robinson (RGAR) loss scores

**Figure S2 - Matrix visualization for expression profiles map with**

**corresponding pair-wise correlation map for Yeast cell cycle data [2]**

Matrix visualization for expression profiles map with corresponding pair-wise correlation matrix map of 145 cell cycle related genes [2] with eight sorting algorithms.

(a) SVD1; (b) SVD2; (c) SOM order; (d) R2E seriation;

(e) HCT_RAM order; (f) HCT_OPT order; (g) HCT_SOM order;

(h) HCT_R2E order;

**Figure S3 - Generalized anti-Robinson loss scores for Yeast cell cycle data [2]**

Loss scores with varying window-sizes 145 cell cycle related genes for eight sorting algorithms: SVD1, SVD2, SOM, R2E, HCT_RAM, HCT_OPT, HCT_SOM, and HCT_R2E.

(a) Generalized anti-Robinson (GAR) loss scores;
(b) Relative generalized anti-Robinson (RGAR) loss scores.

**Figure S4 – Cell cycle phase diagrams for** **Yeast cell cycle data [2]**

Matching scores of the rearranged phase positions of the 145 genes sorted by the eight seriation methods to the known (annotated) phase positions.

(a) SVD1; (b) SVD2; (c) SOM order; (d) R2E seriation;

(e) HCT_RAM order; (f) HCT_OPT order; (g) HCT_SOM order;

(h) HCT_R2E order.

**Figure S5 - Matrix visualization for Severe acute respiratory syndrome coronavirus (SARS-CoV) data [3]**

Matrix visualization for expression profiles map with corresponding pair-wise Euclidean distance map of 55 samples in the SARS study [3] with eight sorting algorithms.

(a) SVD1; (b) SVD2; (c) SOM order; (d) R2E seriation;

(e) HCT_RAM order; (f) HCT_OPT order; (g) HCT_SOM order;

(h) HCT_R2E order.

**Figure S6 - Generalized anti-Robinson loss scores for Severe acute respiratory syndrome coronavirus (SARS-CoV) data [3]**

Loss scores with varying window-sizes for c corresponding pair-wise Euclidean distance map of 55 samples in the SARS study with eight sorting algorithms: SVD1, SVD2, SOM, R2E, HCT_RAM, HCT_OPT, HCT_SOM, and HCT_R2E.

(a) Generalized anti-Robinson (GAR) loss scores;
(b) Relative generalized anti-Robinson (RGAR) loss scores.

**Figure S7 - The rank-two ellipse (R2E) converging sequence of correlation matrices**

The rank-two ellipse (R2E) converging process for the 517*517 between-gene correlation matrix of the fibroblasts to serum data.

**Figure S8 – Java GAP working environment with SARS-CoV data [3]**

GAP has a standard multi-window operating environment. This figure demonstrates the GAP GUI using SARS Dataset [3]. (a) The unsorted raw data map and two proximity maps with clinical records on sample group, on the left; (b) HCT_RAM order; (c) R2E seriation; (d) HCT_R2E order.
